# Sleep architecture and rapid eye movement sleep without atonia in post-COVID-19 insomnia

**DOI:** 10.1093/sleep/zsaf257

**Published:** 2025-09-16

**Authors:** Abubaker Ibrahim, Matteo Cesari, Qi Tang, Merve Aktan Süzgün, Elisabeth Brandauer, Evi Holzknecht, Alexander Wachter, Victoria Anselmi, Anna Heidbreder, Ambra Stefani, Birgit Högl

**Affiliations:** Department of Neurology, Medical University of Innsbruck, Innsbruck, Austria; Department of Neurology, Medical University of Innsbruck, Innsbruck, Austria; Department of Neurology, Medical University of Innsbruck, Innsbruck, Austria; Department of Neurology, Medical University of Innsbruck, Innsbruck, Austria; Department of Neurology, Medical University of Innsbruck, Innsbruck, Austria; Department of Neurology, Medical University of Innsbruck, Innsbruck, Austria; Department of Neurology, Medical University of Innsbruck, Innsbruck, Austria; Department of Neurology, Medical University of Innsbruck, Innsbruck, Austria; Department of Neurology, Johannes Kepler University Linz, Linz, Austria; Department of Neurology, Medical University of Innsbruck, Innsbruck, Austria; Department of Neurology, Medical University of Innsbruck, Innsbruck, Austria

**Keywords:** COVID-19, insomnia, long-COVID, polysomnography, REM sleep without atonia

## Abstract

**Study Objectives:**

Insomnia associated with COVID-19 infection is a common complaint in long-COVID. Studies to date have predominantly examined post-COVID-19 sleep disturbances with questionnaires. We aimed to investigate whether there are distinctive polysomnographic findings in post-COVID-19 insomnia compared to non-COVID-related chronic insomnia.

**Methods:**

We included 150 patients with chronic insomnia, stratified into three groups: post-COVID-19 insomnia (*N* = 50), chronic insomnia during the pandemic without a history of COVID-19 infection (*N* = 50), and pre-pandemic chronic insomnia (*N* = 50). All patients underwent one-night video-polysomnography (v-PSG). The sleep architecture, respiratory variables, and rapid eye movement (REM) sleep without atonia (RWA) were compared across the groups.

**Results:**

Classical polysomnographic variables showed no significant differences across groups with regard to total sleep time, sleep efficiency, sleep stage percentages, and the apnea–hypopnea index. Post-COVID-19 insomnia patients had significantly increased RWA at both the chin and the flexor digitorum superficialis (*p=*.020 for both), and higher nocturnal heart rates (*p=*.046). Sleep-bout analysis indicated shorter sustained N3-sleep periods (*p=*.001) and longer onset to stable REM sleep (*p=*.016) in the post-COVID-19 insomnia group. Although sleep transitions did not withstand multiple comparison corrections, they revealed a trend toward decreased N3-sleep continuity and increased probabilities of transitioning to lighter stages (N3 → N3: *unadjusted*-*p=*.012; REM → N1: *unadjusted*-*p=*.027) in the post-COVID-19 insomnia.

**Conclusions:**

Classical PSG profile of post-COVID-19 insomnia does not differ from non-COVID-related chronic insomnia. However, subtle differences in RWA and sleep integrity suggest that post-COVID-19 insomnia is driven not merely by pandemic-related stress factors but by additional physiological alterations linked to viral central nervous system involvement.

**Statement of Significance**

Insomnia is a major symptom in long-COVID, yet objective studies examining its electrophysiological underpinnings are scarce. We present the largest video-polysomnographic investigation comparing post-COVID-19 insomnia with chronic insomnia unrelated to COVID-19. While classical sleep architecture metrics did not significantly differ between post-COVID-19 insomnia and matched pre-pandemic and pandemic-era non-COVID-insomnia patients, our findings reveal subtle but important distinctions. Patients with post-COVID-19 insomnia exhibited significantly increased REM RWA, alongside trends toward altered sleep continuity, including shorter N3 sleep bouts, longer onset to stable REM sleep, and higher nocturnal heart rates. These findings suggest specific central nervous system alterations post-infection that go beyond stress-related pandemic effects and are likely linked to viral central nervous system effects. This highlights the unique neurobiological impact of COVID-19 on sleep regulation, meriting further investigation, even as established insomnia treatments remain applicable.

## Graphical Abstract

**Graphical Abstract:**
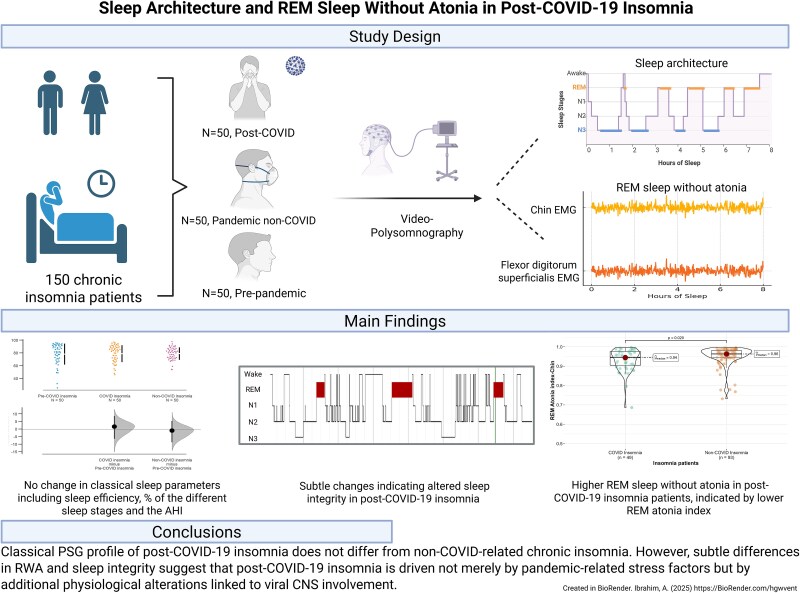

## Introduction

The COVID-19 pandemic has introduced unprecedented challenges to health systems around the world, with far-reaching implications for mental and physical well-being. Among these effects, sleep disruptions have emerged as a critical concern, with insomnia becoming a widespread issue [1]. Two meta-analyses have shown a pooled prevalence of COVID-19-related insomnia between 23.8 per cent and 52.5 per cent among affected individuals [2, 3]. This surge is thought to be driven by a combination of heightened stress, anxiety, and depression, as well as disruptions to daily routines and exposure to blue light from increased screen time [4, 5]. Insomnia can affect individuals exposed to COVID-19, regardless of the severity of their initial symptoms, and may continue long after the acute phase of the infection has resolved [6, 7]. A recent multicenter study (International Covid Sleep Study Collaboration, ICOSS-II) showed that fatigue, insomnia symptoms, and excessive daytime sleepiness were highly prevalent among respondents reporting long-lasting symptoms after COVID-19 [8]. Moreover, in a cohort of 42 patients seen at a dedicated post-COVID-19 sleep clinic, at least five sleep disorders were prevelant: obstructive sleep apnea (OSA, 35.7 per cent), chronic insomnia (28.6 per cent), primary hypersomnia (21.4 per cent), rapid eye movement (REM) sleep behavior disorder (RBD, 11.9 per cent), and circadian disturbances (4.8 per cent) [9].

According to the International Classification of Sleep Disorders (ICSD-3-TR), chronic insomnia is characterized by difficulty initiating or maintaining sleep and/or non-restorative sleep with clinically significant distress, occurring at least three nights per week for more than 3 months, and resulting in daytime dysfunction [10]. Insomnia is diagnosed on the basis of clinical history. Nonetheless, video-polysomnography (v-PSG) can be helpful in evaluating patients with insomnia symptoms when there is suspicion of comorbid sleep disorders (e.g. sleep-related breathing disorders) [11, 12]. Previous studies showed that insomnia with objectively measured short sleep duration (<6 h) identified through v-PSG is associated with cognitive impairment, systemic inflammation, hypertension, and incident cardiovascular/cerebrovascular diseases [13–16]. Insomnia patients have increased arousal [17], as evidenced by high-frequency electroencephalography (EEG) activity, increased microarousal, and REM sleep instability [17–19]. A recent study has demonstrated that those suffering from chronic insomnia have a higher probability of transitioning from stage N2- to stage N1-sleep or wakefulness, indicating specific N2 vulnerability in these patients [20].

It is unknown whether COVID-19-related insomnia is associated with polysomnographic findings that diverge from those of patients with non-COVID-related chronic insomnia. To date, there have been no large v-PSG studies on chronic insomnia after COVID-19 infection. Only one polysomnographic study in 17 post-COVID-19 insomnia patients showed a slightly lower N3-sleep percentage in post-COVID-19 insomnia patients compared to insomnia not associated with COVID-19 [21]. Additionally, we previously described higher REM sleep without atonia (RWA) in 11 post-COVID-19 patients (with and without insomnia), but this was never investigated in a large cohort [22].

Insomnia that emerged during the pandemic, even without a history of COVID-19 infection, may reflect the influence of widespread stressors such as social isolation, uncertainty, and disrupted routines. Comparing this group to pre-pandemic and post-COVID-19 insomnia could clarify the role of pandemic-specific factors versus infection-related factors.

We sought to investigate v-PSG findings in a large cohort of patients with (i) post-COVID-19 insomnia and compare them to two matched patient cohorts with chronic insomnia without a history of COVID-19 infection, (ii) during, and (iii) before the pandemic.

## Materials and Methods

### Participants

We conducted a retrospective analysis of polysomnographies from patients diagnosed with chronic insomnia, stratified into three groups: (i) post-COVID-19 insomnia: those with chronic insomnia whose symptoms began during or immediately after a COVID-19 infection and persisted at least 3 months after infection (*N* = 50); (ii) pandemic-era non-COVID insomnia: age- and sex-matched chronic insomnia controls without a clinical history of COVID-19 infection, with symptoms starting during the pandemic (January 2020–January 2023) (*N* = 50); (iii) pre-COVID-insomnia: age- and sex-matched chronic insomnia controls diagnosed before the pandemic (prior to December 2019) (*N* = 50). In total, 150 patients were included in the study. Inclusion criteria included the diagnosis of chronic insomnia according to the third edition of the International Classification of Sleep Disorders (ICSD-3) [23]. For COVID-19 insomnia cases, v-PSG was conducted 3–24 months after an acute COVID-19 infection.

Participants were selected consecutively from patients diagnosed with chronic insomnia who underwent v-PSG (starting in 2023 and backward). They were matched according to age and sex. Seven patients from the cohort reported by Heidbreder et al. [22], were included in the present study as part of the post-COVID-19 group.

This study has been conducted in accordance with the Declaration of Helsinki and was approved by the local ethics committee.

### Polysomnography

All patients underwent one night of v-PSG at the Center of Sleep Medicine, Department of Neurology, Medical University of Innsbruck, Austria. v-PSG was performed according to the most current guidelines of the American Academy of Sleep Medicine (AASM) [24] at the time of v-PSG. Recorded channels included horizontal electrooculography, six-channel EEG, surface electromyography (EMG) as recommended by the Sleep Innsbruck Barcelona (SINBAR) montage of the mental, submental, both flexor digitorum superficialis (FDS), and anterior tibialis muscles, cardiorespiratory monitoring (electrocardiography, oronasal airflow, and thermal sensor; tracheal microphone; oxygen saturation (SPO_2_); thoracic and abdominal respiratory movements), and time-synchronized digital videography. Sleep stages and respiratory events were manually scored according to the AASM criteria [24]. Periodic leg movements during sleep were calculated according to the AASM criteria using a validated software [25].

All participants had received a clinical diagnosis of chronic insomnia disorder prior to undergoing v-PSG. The decision to perform v-PSG was based on persistent symptoms and the need to identify, treat, or exclude potential comorbid sleep disorders, given the high comorbidity between insomnia and other sleep conditions [11, 12]. In addition, many participants reported sleep maintenance difficulties, warranting further investigation. In the post-COVID-19 insomnia group, v-PSG was also conducted due to the temporal association with previous SARS-CoV-2 infection; given the known impact of COVID-19 on pulmonary functions, it was important to assess possible sleep-related breathing disturbances. Finally, in our clinical routine, v-PSG is often used to evaluate possible sleep state misperception.

### Classical sleep variables

From v-PSG we obtained time in bed, total sleep time (TST), sleep efficiency, sleep latency, sleep period time (SPT), duration and percentages of the different sleep stages in SPT, apnea-hypopnea index (AHI) during sleep, average oxygen saturation during the night as well as desaturation events ≥4 per cent. We calculated arousal indices using a validated algorithm [26].

### Sleep stage shifts and sleep fragmentation

We calculated the empirical probability of transitions between stages for each patient. For example, we calculated the empirical probability of transitions between wake (W) and N1 sleep as the number of transitions between W and N1 divided by the total number of transitions between W and any other stage (including W itself). This was also done for all the sleep stages and transitions, as done following a previous study [20]. We excluded from statistical analyses rare empirical transition probabilities, i.e. those not present in fewer than 50 per cent of the total cohort. Additionally, we calculated the sleep fragmentation index (SFI) as the total number of shifts to N1 sleep (from NREM or REM sleep) divided by the TST/h [27]. We also measured a modified SFI, including any sleep stage shift and the total number of awakenings, divided by TST/h [28].

### Measurement of sleep bouts and onset to stable sleep

Sleep bouts were identified as continuous periods within a specific sleep stage (N1, N2, N3, REM, or W). We counted the total number of bouts, the average duration, and the duration of the longest bout in each stage. Onset to stable sleep was defined as the time from the light off to the first epoch of any sustained sleep stage, lasting at least 10 consecutive minutes. For N2, N3, and REM sleep, we additionally calculated the time from the first epoch of sleep to the first epoch of a sustained sleep stage, lasting at least 10 consecutive minutes.

### REM sleep without Atonia

In order to assess REM sleep without atonia, we used the REM atonia index (RAI) [29, 30] on EMG signals recorded from the chin and the average of the FDS muscles (right and left). This index can vary from 0, which means the complete absence of EMG atonia, to 1, meaning complete EMG atonia [29]. We performed manual artifact inspection before signal analysis; here, if the channel/channels were not usable or completely missing, we excluded the recording from the analysis. Altogether, seven recordings had to be excluded from RWA analysis for FDS, and eight for the chin (FDS: one post-COVID-19, two pandemic-era non-COVID, four pre-pandemic insomnia; chin: one post-COVID-19, three pandemic-era non-COVID, four pre-pandemic insomnia), with no significant group differences (Fisher’s exact test: chin *p*=.535; FDS *p*=.507).

### Statistics

Statistical analyses were performed with R version 4.1.2 (R Foundation for Statistical Computing, Vienna, Austria) and IBM SPSS Statistics V.26 for Windows (IBM Corp., Armonk, NY, USA). Data distributions were analyzed using the Shapiro–Wilk test. Variables were predominantly non-normally distributed; therefore, summary data are expressed as median (interquartile range, IQR). Data were compared between the three groups (post-COVID-19 insomnia versus pre-COVID-insomnia versus pandemic-era non-COVID insomnia). Additionally, we compared post-COVID-19 insomnia versus non-COVID-related insomnia (combining insomnia before and during the pandemic without COVID-19 in the clinical history) and pre- versus post-pandemic (combining post-COVID-19 insomnia and pandemic-era non-COVID insomnia). The Mann–Whitney *U* test and the Kruskal–Wallis test were used to compare numerical variables. Pearson chi-square and Fisher’s exact tests were used for categorical variables. The 95% confidence interval around the estimated median difference of macro-sleep variables within the different groups was estimated via bootstrap resampling (set to 5000 samples) and visualized with the Gardner–Altman plot [31]. The sleep stage transition probability was visualized with a Sankey diagram [32]. Here, for better visualization, we excluded the same-stage transition probability.

The rank-based effect size (ES) was computed with the “**rstatix**” package as the standardized test statistic (*Z*) divided by the square root of the total sample size [33]. Two-tailed *p*-values <0.05 were considered statistically significant. Adjustment for multiple comparisons when analyzing the sleep transitions was made using the Benjamini–Hochberg procedure. However, we also chose to report non-adjusted *p*-values due to the exploratory nature of the study. The interpretation values for the ES are as follows: 0.10 − <0.3 (small effect), 0.30 − <0.5 (moderate effect), and ≥0.5 (large effect).

## Results

### Demographic and clinical characteristics

Table 1 shows the demographics and medication comparison among the three groups. There was no significant difference in age, sex, BMI, or medication use across the three groups. Regarding other sleep comorbidities 13/150 patients were diagnosed with restless legs syndrome (5 [10 per cent] in the post-COVID-19 insomnia and 8 [8 per cent] in the non-COVID-related insomnia group), 9/150 were diagnosed with sleep-related bruxism (2 [4 per cent] in the post-COVID-19 insomnia group and 7 [7 per cent] in the non-COVID-related insomnia group), and 32/150 with sleep-related breathing disorder (11 [22 per cent] in the post-COVID-19 insomnia group and 21 [21 per cent] in the non-COVID-related insomnia). The breathing disorders were further classified as 24 mild cases (AHI 5–15 ev/h), 7 moderate cases (AHI >15–30 ev/h), and 1 severe case (AHI >30 ev/h). None of these differences were statistically significant across the groups. There were no other comorbid sleep disorders. There was no difference in the Epworth Sleepiness Scale (ESS) among the groups.

**Table 1:** Demographics and medication information

**Variable**	**COVID-19 insomnia, *N* = 50**	**Insomnia pre-pandemic, *N* = 50**	**Pandemic-era Non-COVID insomnia, *N* = 50**	***p*-value**
**Age**	48.9 (37.0–56.7)	49.6 (33.2–55.8)	53.6 (45.1–57.8)	0.258
**Sex (f/m)**	25/25	25/25	25/25	-
**BMI**	25.9 (23.0–27.7)	23.0 (21.3–25.2)	24.2 (21.3–27.4)	0.05
**ESS**	4 (2–7)	4 (2–7)	3 (1–5.7)	0.403
**Medications, *N* (%)**				
Antidepressants^*^	22 (44)	15 (30)	14 (28)	0.229
**Neuroleptics**	1 (2)	3 (6)	4 (8)	0.534
**Benzodiazepines**	2 (8)	2 (4)	3 (4)	1
**Hypnotics**^†^	2 (4)	3 (6)	6 (12)	0.386
**Antihypertensive medications**	7 (14)	9 (18)	7 (14)	0.879
**Beta-blockers**^‡^	6 (12)	5 (10)	2 (4)	0.431
**Thyroid substitution therapy**	6 (12)	6 (12)	10 (20)	0.458

Continuous variables are shown as median and interquartile range (IQR) Mann–Whitney *U* tests was used to compare significance. Categorical variables are shown as percentages and analyzed with chi-squared test. *Abbreviations*: BMI, body mass index; COVID, coronavirus disease; ESS, Epworth Sleepiness Scale; f/m: female/male patients.

^*^Sixteen patients took antidepressants because of an affective mood disorder, 26 as a sleep aid, for 9 patients the reason is unknown.

^†^This group includes the Z drugs and Hetrazepine (heterocyclic antidepressants).

^‡^Beta-blockers are separated from other antihypertensive drugs since the indication is not always hypertension.

### Classical sleep variables

**Figure 1:**
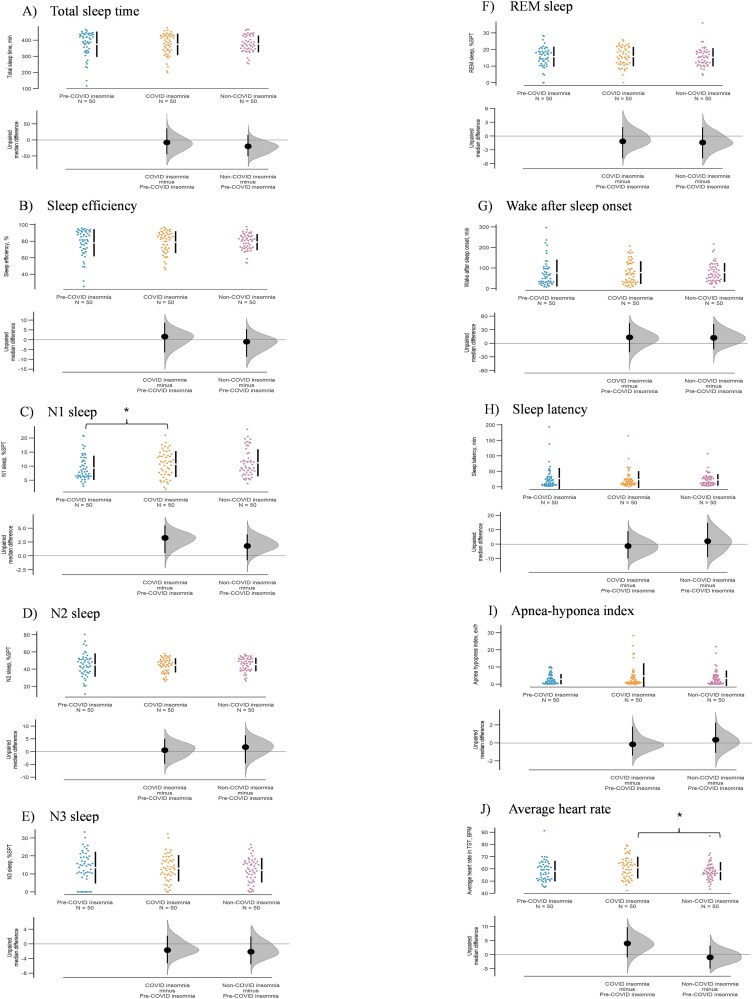
Gardner–Altman plots comparing sleep architecture and respiratory variables across pre-pandemic, post-COVID-19, and non-COVID (pandemic-era without clinical history of COVID-19) groups, with effect sizes and 95% confidence intervals shown.

The sleep structure variables and respiratory parameters are presented in Table 2. Sleep architecture variables, including TST, sleep efficiency, sleep onset, REM sleep onset, and the percentage of the different sleep stages in SPT, showed no significant differences between the groups (all *p* > .05). When comparing post-COVID-19 insomnia versus all non-COVID insomnia cases (i.e. pre and post-pandemic, *N* = 100) as shown in Table 3, the average heart rate during the night was significantly higher in post-COVID-19 insomnia patients, *p =* .046. Regarding the respiratory parameters, although the apnea–hypopnea index showed no significant difference across the groups, the mean oxygen saturation was lower in post-COVID-19 insomnia patients compared to pre-pandemic insomnia, *p =* .028. The mean differences (95% confidence interval) in selected architecture and respiratory parameters are illustrated in Figure 1 (Gardner–Altman plot).

**Table 2:** Polysomnographic sleep variables in insomnia patients

**Variable**	**COVID-19 insomnia, *N* = 50**	**Insomnia pre-pandemic, *N* = 50**	**Pandemic-era** **Non-COVID insomnia, *N* = 50**	**COVID-19 vs. pre** ***P*-value ES**		**Pre- vs. Pan-non** ***P*-value ES**		**COVID-19 vs. Pan-non** ***P*-value ES**		**Kruskal–Wallis**
Time in bed, min	479.5 (464.5–489.8)	473.5 (467.0–482.8)	482.5 (467.2–495.0)	.426	.08	.152	.14	.442	.08	0.333
Total sleep time, min	385.5 (335.2–426.5)	393.0 (334.2–433.8)	373.5 (343.5–426.8)	.610	.05	.528	.06	.970	.00	0.800
Sleep efficiency, %	83.2 (69.8–89.0)	81.6 (70.6–91.3)	80.6 (72.0–86.1)	.788	.03	.461	.07	.603	.05	0.741
Sleep latency, min	15.4 (8.0–25.4)	16.6 (6.8–31.5)	18.7 (9.0–30.6)	.981	.00	.654	.05	.539	.06	0.824
REM latency, min	86.0 (70.0–151.5)	105.5 (81.4–171.2)	103.2 (72.8–159.1)	.217	.13	.384	.09	.693	.04	0.442
N1 sleep, %SPT	11.2 (7.0–14.0)	7.9 (6.3–12.0)	9.7 (7.8–14.1)	.092	.17	**.047**	.20	.844	.02	0.101
N2 sleep, %SPT	45.6 (38.1–50.6)	45.1 (36.5–51.7)	46.9 (39.5–51.7)	.983	.00	.556	.06	.524	.06	0.775
N3 sleep, %SPT	12.8 (9.1–17.6)	14.5 (9.0–19.3)	12.3 (6.8–16.6)	.743	.03	.438	.08	.542	.06	0.696
REM sleep, %SPT	15.5 (11.4–21.0)	16.6 (12.2–18.9)	15.2 (11.6–18.5)	.893	.01	.478	.07	.436	.08	0.683
Wake after sleep onset, min	69.3 (31.3–122.5)	56.1 (29.1–97.3)	68.3 (40.1–104.7)	.584	.06	.173	.14	.647	.05	0.457
Wake, %SPT	12.6 (6.6–25.1)	12.2 (5.8–23.6)	14.7 (8.6–22.9)	.793	.03	.292	.11	.488	.07	0.572
Arousal index in TST, ev/h	18.3 (12.5–22.1)	15.9 (12.8–20.4)	16.6 (11.7–23.8)	.260	.11	.272	.11	.920	.01	0.433
Apnea–hypopnea index, ev/h	1.6 (0.6–4.8)	1.7 (0.5–4.7)	2.0 (0.3–4.8)	.725	.04	.912	.01	.692	.04	0.905
Apnea index, ev/h	0.3 (0.0–0.7)	0.3 (0.1–0.7)	0.2 (0.0–0.6)	.735	.03	.460	.07	.803	.03	0.785
Hypopnea index, ev/h	1.1 (0.3–3.8)	1.2 (0.2–3.0)	1.4 (0.2–3.8)	.636	.05	.717	.04	.907	.01	0.882
Oxygen desaturation index >4%, %	1.3 (0.3–4.1)	0.9 (0.2–2.2)	1.4 (0.3–3.6)	.140	.15	.136	.15	.986	.00	0.228
Mean oxygen saturation, %	94.9 (93.9–95.6)	95.3 (94.4–96.6)	94.8 (94.1–95.9)	**.028**	.22	.069	.18	.684	.04	0.063
Minimal oxygen saturation, %	89.5 (86.0–91.4)	89.0 (86.0–91.8)	89.0 (87.0–91.0)	.753	.03	.681	.04	.784	.03	0.893
PLMS index, ev/h	6.6 (0.8–19.6)	3.6 (0.3–9.1)	4.8 (1.4–16.5)	.162	.14	.177	.14	.890	.01	0.280
Average heart rate in TST, BPM	62.0 (53.5–66.3)	58.0 (51.4–63.2)	57.0 (54.1–60.9)	.088	.17	1.000	.00	.083	.17	0.137

Values are shown as median and (interquartile range). Mann–Whitney *U* tests were used to compare the groups. Significant *p* values are marked in bold. *Abbreviations*: AHI, apnea–hypopnea index; BPM, beats per minute; COVID, coronavirus disease; ES, effect size; ev/h, events per hour; ODI, oxygen desaturation index; PLMS, periodic leg movements during sleep; REM, rapid eye movement; SPT, sleep period time; TST, total sleep time.

**Table 3:** Polysomnographic sleep variables in COVID-19 Insomnia vs. non-COVID-related Insomnia patients

**Sleep feature**	**COVID-19 insomnia, *N* = 50**	**Non-COVID-related insomnia, *N* = 100**	***P* value**	**Effect size**
Time in bed, min	479.5 (464.5–489.8)	477.0 (467.0–490.5)	.989	0
Total sleep time, min	385.5 (335.3–426.5)	384.0 (335.8–429.0)	.786	0.02
Sleep efficiency, %	83.3 (69.8–89.0)	81.4 (71.8–88.6)	.886	0.01
Sleep latency, min	15.4 (8.0–25.4)	17.1 (7.4–30.9)	.711	0.03
REM latency, min	85.3 (68.5–146.8)	108.0 (76.9–181.6)	.349	0.08
N1 sleep, %SPT	11.2 (7.0–14.0)	9.1 (6.5–12.9)	.391	0.07
N2 sleep, %SPT	45.7 (38.2–50.6)	46.2 (38.3–51.8)	.723	0.03
N3 sleep, %SPT	12.8 (9.1–17.7)	13.1 (8.0–17.7)	.872	0.01
REM sleep, %SPT	15.5 (11.4–21)	15.5 (11.9–18.8)	.596	0.04
Wake after sleep onset, min	69.4 (31.3–122.5)	65.5 (34.1–103.5)	.96	0
Wake, %SPT	12.6 (6.6–25.1)	13.8 (7.4–23.4)	.805	0.02
Arousal index TST	16.5 (12.3–21.1)	18.3 (12.5–22.1)	.554	0.05
Apnea–hypopnea index, ev/h	1.6 (0.7–4.8)	1.9 (0.4–4.9)	.664	0.04
Apnea index, ev/h	0.3 (0–0.7)	0.3 (0.1–0.7)	.96	0
Hypopnea index, ev/h	1.2 (0.3–3.8)	1.3 (0.2–3.2)	.731	0.03
Oxygen desaturation index > 4%, %	1.3 (0.3–4.1)	1.3 (0.3–3.1)	.387	0.07
Mean oxygen saturation, %	94.9 (93.9–95.6)	95.2 (94.2–96.2)	.133	0.12
Minimal oxygen saturation, %	89.5 (86.0–91.4)	89.0 (86.0–91.0)	.981	0
PLMS index, ev/h	6.6 (0.8–19.6)	4.3 (0.8–10.3)	.374	0.07
Average heart rate in TST, BPM	62.1 (53.5–66.4)	57.1 (52.3–62.4)	**.046**	0.16

Values are shown as median and (interquartile range). Mann–Whitney *U* tests were used to compare the groups. Significant *p* values are marked in bold. *Abbreviations*: AHI, apnea–hypopnea index; COVID, coronavirus disease; ES, effect size; ev/h, events per hour; ODI, oxygen desaturation index;PLMS, periodic leg movements during sleep; REM, rapid eye movement; SPT, sleep period time; TST, total sleep time.

### Sleep stage shifts and sleep fragmentation

The analysis of sleep stage transitions is shown in Table 4. When considering the same-stage transition, the transition probability from N3 to N3 (i.e. continuity of N3) was different across the group (*p =* .012, unadjusted), with the lowest transition probability found in the COVID-19 insomnia group. When considering transitioning into a different stage, the post-COVID-19 insomnia group had a significantly higher probability of transitioning from REM to N1 sleep and vice versa (*p =* .027, *p =* .029, both unadjusted) compared to pre-pandemic insomnia. When comparing post-COVID-19 insomnia versus all non-COVID-related insomnia, as shown in Table S1, the transition probabilities from N2 sleep to Wake and REM to N2 sleep were significantly higher in the non-COVID-related insomnia group compared to the post-COVID-19 insomnia group (*p=*.007, *p=*.027, both unadjusted). After correction for multiple comparisons, none of the *p-*values remained significant. A visual presentation of the mean sleep transition probability in the three groups, excluding the same-stage transition, is shown in Figure S1.

The SFI (Haba-Rubio) was significantly lower in the pre-pandemic insomnia compared to post-pandemic insomnia, regardless of COVID-19 infection status: 13.8/h (11.1/h–17.8/h) versus 16.5/h (13.3/h–22.2/h), *p =* .027.

### Sleep bout and onset to stable sleep

The results of the sleep bouts and onset to stable sleep are shown in Table 5. The average and longest N3 sleep and REM duration bouts were different across the groups (*p* = .014, *p* = .001, *p* = .027), with the shortest median being in post-COVID-19 insomnia. The pre-pandemic insomnia had a shorter N1 sleep bout compared to post-pandemic insomnia, regardless of COVID-19 infection status (*p=*.017). When comparing post**-**COVID-19 insomnia versus all non-COVID-related insomnia (Table S2), the onset to stable REM sleep was significantly longer in the post-COVID-19 insomnia compared to the non-COVID-related insomnia (*p =* .016).

### REM sleep without Atonia

**Figure 2:**
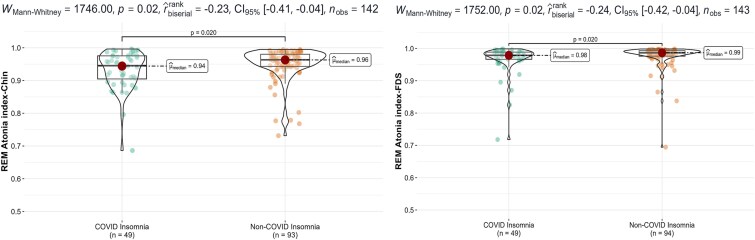
Comparison of REM sleep without atonia (RWA) as measured with the REM atonia index (RAI) between post-COVID-19 and non-COVID-related insomnia groups shown as box/violin plots for the chin (A) and flexor digitorum superficialis (FDS) (B), with the median represented by a dot and the shape outline indicating the kernel density estimate.

**Figure 3:**
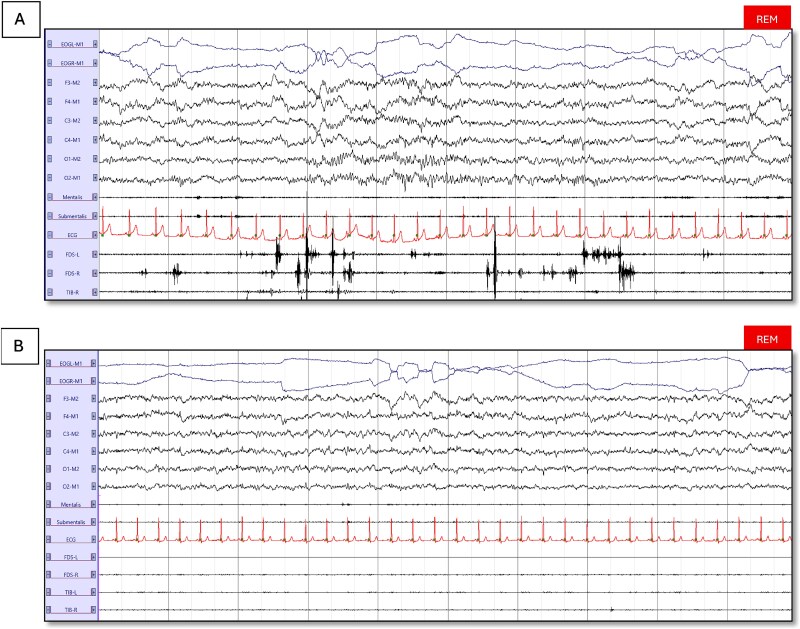
Representative 30-second polysomnography epochs from a post-COVID insomnia patient (A) and a non-COVID-related insomnia patient (B), illustrating REM sleep without atonia in (A) with EMG bursts in the flexor digitorum superficialis, and physiological REM atonia in (B). Abbreviations: ECG, electrocardiography; EOG, electrooculography; FDS-L/R, flexor digitorum superficialis left and right; Tib-L/R, tibialis anterior left and right.

In the post-COVID-19 insomnia group (*n* = 49), both REM atonia indices—RAI-Chin and RAI-FDS—were significantly lower than in the combined non-COVID insomnia cohort (*n* = 93/94; RAI-Chin *p*=.020, ES = 0.23; RAI-FDS *p*=.020, ES = 0.24; see Fig. 2). The difference became even more pronounced when we compared the the pre-pandemic insomnia group (*N* = 46) directly with the post-COVID-19 group: median RAI-Chin dropped from 0.97 (0.94–0.98) to 0.94 (0.89–0.97) (*p* = .014, ES = 0.25), and RAI-FDS from 0.98 (0.97–0.99) to 0.97 (0.96–0.98) (*p*=.006, ES = 0.28). None of the patients met the ICSD-3 criteria for RBD. A representative tracing of RWA versus normal REM atonia is shown in Fig. 3.

**Table 4:** Stage shifts and transition probabilities between sleep stages

**Variable**	**COVID-19 insomnia, *N* = 50**	**Insomnia pre-pandemic, *N* = 50**	**Pandemic-era** **Non-COVID insomnia, *N* = 50**	**COVID-19 vs. Pre**		**Pre vs. Pan-non**		**COVID-19 vs. Pan-non**		**Kruskal–Wallis**
				***P* value ES**		***P* value ES**		***P* value ES**		
**Wake → Wake**	85.5 (76.6–93.1)	85.7 (79.7–91.4)	84.9 (81.1–89.4)	.807	.02	.754	.03	.637	.05	0.879
**Wake → N1 sleep**	13.5 (6.2–23.1)	12.7 (8.5–20.1)	15.1 (10.4–18.9)	.801	.03	.652	.05	.493	.07	0.779
**Wake → N2 sleep**	0.0 (0.0–0.0)	0.0 (0.0–0.5)	0.0 (0.0–0.0)	-	-	-	-	-	-	-
**Wake → N3 sleep**	0.0 (0.0–0.0)	0.0 (0.0–0.0)	0.0 (0.0–0.0)	-	-	-	-	-	-	
**Wake → REM sleep**	0.0 (0.0–0.0)	0.0 (0.0–0.0)	0.0 (0.0–0.0)	-	-	-	-	-	-	-
**N1 sleep → Wake**	8.0 (4.3–11.9)	9.1 (4.8–12.4)	9.3 (5.9–12.4)	.574	.06	.57	.06	.196	.13	0.461
**N1 sleep → N1 sleep**	56.2 (47.3–67.9)	57.0 (46.5–64.8)	56.2 (46.8–62.2)	.634	.05	.657	.04	.266	.11	0.573
**N1 sleep → N2 sleep**	27.6 (21.6–38.0)	33.5 (25.8–38.8)	30.0 (21.9–38.7)	.2	.13	.617	.05	.408	.08	0.42
**N1 sleep → N3 sleep**	0.0 (0.0–0.0)	0.0 (0.0–0.0)	0.0 (0.0–0.0)	-	-	-	-	-	-	-
**N1 sleep → REM sleep**	2.6 (0.0–6.0)	1.5 (0.0–2.6)	2.1 (0.1–6.3)	**.029**	.22	.078	.18	.986	0	0.073
**N2 sleep → Wake**	2.0 (1.1–2.7)	1.9 (1.6–3.2)	2.7 (2.0–3.6)	.137	.15	.091	.17	**.001**	.32	**0.006**
**N2 sleep → N1 sleep**	2.8 (1.1–4.6)	2.2 (1.3–3.9)	2.9 (1.6–4.8)	.491	.07	.224	.12	.667	.04	0.486
**N2 sleep → N2 sleep**	93.1 (90.9–95.3)	92.7 (90.8–94.6)	91.7 (90.2–93.8)	.983	0	.114	.16	.139	.15	0.207
**N2 sleep → N3 sleep**	1.2 (0.6–1.9)	1.0 (0.6–1.6)	1.1 (0.5–1.6)	.218	.12	.664	.04	.501	.07	0.484
**N2 sleep → REM sleep**	0.0 (0.0–0.0)	0.0 (0.0–0.0)	0.0 (0.0–0.0)	-	-	-	-	-	-	-
**N3 sleep → Wake**	1.0 (0.0–1.9)	1.2 (0.9–2.0)	1.1 (0.0–2.4)	.209	.13	.402	.09	.732	.04	0.453
**N3 sleep → N1 sleep**	0.0 (0.0–1.0)	0.0 (0.0–0.0)	0.0 (0.0–0.9)	-	-	-	-	-	-	-
**N3 sleep → N2 sleep**	2.3 (1.2–3.3)	1.5 (0.6–2.5)	2.0 (0.7–3.0)	.063	.2	.157	.15	.733	.04	0.162
**N3 sleep → N3 sleep**	95.8 (94.4–97.1)	96.9 (96.0–97.5)	95.9 (94.2–97.0)	**.008**	.28	**.011**	.27	.869	.02	**0.012**
**N3 sleep → REM sleep**	0.0 (0.0–0.0)	0.0 (0.0–0.0)	0.0 (0.0–0.0)	-	-	-	-	-	-	-
**REM sleep → Wake**	3.1 (1.5–4.6)	2.3 (1.1–3.7)	2.3 (1.2–4.2)	.156	.14	.68	.04	.329	.1	0.35
**REM sleep → N1 sleep**	1.3 (0.0–3.4)	0.6 (0.0–1.1)	0.8 (0.0–3.1)	**.008**	.27	.054	.2	.618	.05	**0.027**
**REM sleep → N2 sleep**	0.0 (0.0–0.9)	0.8 (0.0–1.2)	0.0 (0.0–1.0)	-	-	-	-	-	-	-
**REM sleep → N3 sleep**	0.0 (0.0–0.0)	0.0 (0.0–0.0)	0.0 (0.0–0.0)	-	-	-	-	-	-	-
**REM sleep → REM sleep**	95.3 (92.3–96.6)	95.8 (94.9–97.0)	94.8 (92.5–96.9)	.059	.19	**.047**	.2	.925	.01	0.081

The values are expressed as median and interquartile range and represent the probability of transitioning between sleep stages as normalized per sleep stage. Same-stage sleep transition indirectly reflects the stability of a certain sleep stage. In bold, the transitions, which are significant before multiple comparison corrections, are highlighted. The statistical comparison between empirical transition probabilities was performed in 16 out of the 25 possible transitions. After multiple comparison correction, none of the comparisons remained significant. *Abbreviations*: COVID, coronavirus disease; ES, effect size; REM, rapid eye movement.

**Table 5:** Onset to stable sleep and sleep bouts

**Variable**	**COVID-19 insomnia** ***N* = 50**	**Insomnia pre-pandemic** ***N* = 50**	**Pandemic-era** **Non-COVID insomnia, *N* = 50**	**COVID-19 vs. pre**		**Pre vs. Pan-non**		**COVID- 19 vs. Pan-non**		**Kruskal–Wallis**
				***P* value ES**		***P* value ES**		***P* value ES**		
**Onset to stable sleep (10 min) after light off, min**	37.2 (20.8–59.9)	35.8 (22.2–69.8)	41.2 (30.2–31.5)	.986	0	.551	.06	.484	.07	0.753
**Onset to stable sleep after SOL (10 min), min**	17.2 (5.9–33.5)	14.0 (7.0–39.9)	22.5 (13.0–36.2)	.863	.02	.383	.09	.366	.09	0.583
**Onset to stable N2 (10 min) after SOL, min**	30.0 (7.0–73.4)	39.8 (12.6–117.1)	39.0 (15.5–90.5)	.303	.1	.933	.01	.314	.64	0.495
**Onset to stable N3 (10min) after SOL, min**	32.0 (19.0–57.0)	34.5 (14.0–64.1)	36.5 (20.2–77.8)	.718	.04	.428	.09	.602	.06	0.707
**Onset to stable REM (10 min) after SOL, min**	190.0 (115.5–283.5)	149.0 (98.9–215.1)	135.2 (88.1–208.8)	.084	.18	.555	.06	**.016**	.25	**0.046**
**Number of N1 bouts**	34.5 (27.0–47.5)	32.0 (23.0–41.8)	42.0 (31.2–54.8)	.277	.11	**.018**	.24	.076	.18	**0.012**
**Number of N2 bouts**	28.0 (20.0–35.0)	26.0 (20.2–35.2)	31.5 (25.2–41.0)	.551	.06	.781	.03	.606	.05	**0.047**
**Number of N3 bouts**	5.0 (3.0–7.0)	4.5 (3.0–6.0)	4.0 (2.0–6.2)	.735	.04	.12	.16	.795	.03	0.854
**Number of REM bouts**	7.0 (5.0–10.0)	6.0 (4.0–7.2)	7.0 (4.0–10.0)	.062	.19	**.018**	.24	.032	.21	0.137
**Number of wake bouts**	21.5 (15.0–31.0)	22.0 (18.0–27.2)	27.0 (21.2–34.0)	.664	.04	.386	.09	**.044**	.2	**0.032**
**Longest N1 duration, min**	5.0 (3.0–6.5)	4.0 (3.0–5.5)	3.8 (2.6–4.5)	.244	.12	.063	.19	.098	.17	0.127
**Longest N2 duration, min**	30.5 (23.6–36.9)	28.8 (24.6–37.4)	24.5 (19.0–35.0)	.981	0	**.001**	.36	.98	0	0.125
**Longest N3 duration, min**	23.0 (16.8–32.5)	29.2 (25.0–41.0)	23.2 (18.0–32.0)	.001^*^	0.36	.049^*^	0.2	.692	.04	0.001^*^
**Longest REM duration, min**	20.5 (16.0–32.5)	27.0 (20.9–38.2)	22.5 (15.6–32.5)	**.009**	.26	.997	0	.71	.04	**0.027**
**Longest wake duration, min**	29.8 (15.0–53.5)	29.0 (18.0–54.5)	30.8 (21.6–46.9)	.697	.04	.632	.05	.251	.12	0.906
**Average N1 bout duration, min**	1.1 (0.9–1.6)	1.2 (0.9–1.4)	1.1 (0.9–1.3)	.634	.05	.112	.16	.143	.15	0.552
**Average N2 bout duration, min**	7.2 (5.5–10.5)	6.9 (5.5–9.3)	6.0 (5.1–8.1)	.967	0	**.013**	.27	0.869	.02	0.208
**Average N3 bout duration, min**	12.0 (9.0–17.2)	15.9 (12.6–18.6)	12.1 (8.7–16.9)	**.008**	.28	.062	.19	.986	0	**0.014**
**Average REM bout duration, min**	10.6 (6.5–14.8)	12.0 (9.6–16.4)	9.6 (6.7–16.1)	.078	.18	.775	.03	.647	.05	0.11
**Average Wake bout duration, min**	3.4 (2.1–6.8)	3.3 (2.4–5.6)	3.1 (2.6–4.6)	.785	.03	.272	.11	.137	.15	0.884
**Sleep fragmentation index (Haba-Rubio)/h**	15.3 (12.4– 19.3)	13.8 (11.1–17.8)	18.1 (14.5–23.4)	.45	.08	**.002**	.31	**.035**	.21	0.068
**Sleep fragmentation index (Morrel)/h**	8.8 (6.7–11.5)	8.5 (6.3–11.0)	10.6 (7.9–15.2)	.754	.03	**.018**	.24	.055	.19	0.119
**Count of transitions to different sleep stages**	94.0 (74.2–131.0)	86.0 (71.8–113.5)	120.0 (94.0–136.0)	.317	.1	**.001**	.34	**.048**	.2	0.068

The values are expressed as median and interquartile range. In bold, the comparisons, which are significant before multiple comparison corrections, are highlighted. The asterisk (^*^) represents significant values which remained significant after multiple comparisons. Onset to stable N1 sleep is not displayed since the median of participants with stable N1 sleep ≥10 min was 0. *Abbreviations*: COVID, coronavirus disease; ES, effect size REM, rapid eye movement; SOL, sleep onset.

## Discussion

In this study, we compared polysomnographic findings in patients diagnosed with chronic insomnia related to COVID-19 versus non-COVID-related insomnia. The study findings reveal no significant differences in classical sleep variables, such as sleep efficiency, latency, the percentages of the sleep stages, and the apnea–hypopnea index between post-COVID-19 insomnia patients and those with non-COVID-related insomnia. However, we found a trend (which did not withstand the correction for multiple comparisons) toward higher probability of sleep stage transitions from REM to lighter sleep stages and less continuity of N3 in the post-COVID-19 group, suggesting more fragmented sleep, in particular during REM and slow wave sleep. Moreover, we found shorter durations of N3 bouts and longer onset to stable REM in the post-COVID-19 group. This is in line with REM-fragmentation described in earlier studies in patients with insomnia [19, 34]. Additionally, we found increased RWA in post-COVID-19 insomnia patients, possibly linked to residual infection-related neurological changes, supporting our previous study [22]. Furthermore, a higher heart rate in COVID-19 insomnia patients may suggest a potential sign of dysautonomia, in line with previous studies showing autonomic dysfunction post-COVID-19 [35, 36]. These findings suggest that post-COVID-19 insomnia is driven not merely by pandemic-related stress factors, but also by additional physiological alterations related to the viral infection itself. Nonetheless, the differences were minor and predominantly aligned with findings found in “conventional” chronic insomnia, thus supporting the treatment of these patients with current established interventions for chronic insomnia.

### Chronic insomnia post-COVID-19

In our study, we included patients with insomnia symptoms persisting beyond the acute COVID-19 infection, fulfilling the diagnostic criteria of chronic insomnia. The global prevalence of COVID-19-related sleep disturbances in patients with long-COVID is estimated to have been about 46 per cent [37]. These disturbances were also reported both acutely post-infection and in hospitalized patients, with prevalence increasing over time [38, 39]. While predisposing factors such as age and sex did not differ in our cohort, we hypothesize that acute viral exposure, persistent viral RNA, and proteins in the central nervous system (CNS), and psychological stress from lockdown measures may have contributed to the onset and persistence of insomnia in post-COVID-19 cases, aligning with the 3P Model of Insomnia [40].

Our findings position these patients within the 2024 National Academies of Sciences, Engineering, and Medicine definition of long-COVID [41]: insomnia emerged within 3 months after infection, persisted at the time of v-PSG, and no alternative explanation was identified. Since we did not administer a comprehensive long-COVID symptom inventory, we cannot determine the prevalence of other post-acute sequelae in this cohort (e.g. fatigue, cognitive impairment). Future work should incorporate validated long-COVID scales to delineate the broader post-viral symptom spectrum and its clinical evolution.

### No changes in classical sleep variables post-COVID-19 insomnia

Studies investigating sleep changes in post-COVID-19 patients have predominantly relied on questionnaires, which generally report poorer sleep quality [42] and higher insomnia severity index (ISI) [3]. Despite recognizing the value of subjective symptoms in insomnia, we focused on objective sleep parameters. In our study, we observed a reduced sleep efficiency in all patients with chronic insomnia (median ≤ 83 per cent in all groups). Importantly, we found no changes in classical sleep parameters in post-COVID-19 insomnia. This is in line with a previous study with 17 patients showing no significant changes in the macro-sleep features compared to matched insomnia patients with no history of long-COVID-19 [21]. Notably, in that study, a slightly reduced N3 sleep percentage in TST was observed in post-COVID-19 insomnia. While the percentage of N3 sleep was not significantly different in our groups, we observed a reduced N3 sleep continuity and significantly shorter N3 bout durations in post-COVID-19 insomnia compared to the other groups. This could partly explain the “brain fog” often reported by post-COVID-19 patients [43], since N3 sleep putatively contributes to next-day cognitive and physical performance and subjective sleep quality [44]. Additionally, recent studies showed increased fatigue and sleepiness in post-COVID-19 patients [8, 45]. In our dataset, we showed that post-COVID-19 insomnia patients did not exhibit systematically higher AHI at a normal/slightly elevated BMI of 25.9 (23.0–27.7) compared to non-COVID-19 insomnia patients. One study showed that 20 post-COVID-19 patients had a higher AHI index compared to healthy controls [46]. However, in that study, the post-COVID-19 patients had a higher BMI at baseline, while we controlled for this confounder. Coelho et al. [9], in a dedicated post-acute sequelae of COVID-19 sleep clinic cohort, found insomnia to be the most common diagnosis after OSA, reinforcing the relevance of our focus. In that study, the OSA patient had an average AHI of 29.3 ± 5.9.

The lack of large differences between pre-pandemic, pandemic-associated, and post-COVID-19 insomnia in the classical sleep variables suggests that the core pathophysiological mechanisms underlying chronic insomnia remain consistent, regardless of external triggers such as the pandemic or COVID-19 infection. This finding indicates that while stressors and contexts may vary, they likely converge on shared pathways of sleep regulation and dysfunction.

### Signs of autonomic dysfunction in post-COVID-19 insomnia patients

We found a higher average heart rate during sleep in COVID-19 insomnia patients when compared specifically to the combined non-COVID insomnia (*p*=.046). This suggests heightened autonomic activity during the night in these patients, potentially contributing to insomnia. Notably, cardiovascular autonomic dysfunction (CVAD) is recognized as a significant component of post-COVID-19 syndrome, affecting approximately one-third of highly symptomatic patients. CVAD manifests through impaired autonomic control of circulatory homeostasis, leading to symptoms such as increased heart rate and blood pressure variability [35, 36]. This autonomic dysregulation is likely a consequence of the viral infection, and its effects on the CNS are also present during sleep.

### Subtle changes in sleep stage transitions and sleep bouts

Our exploratory findings showed a trend toward a higher probability of transitions from REM to N1 sleep in COVID-19 insomnia compared to pre-pandemic insomnia. On the other hand, the transition probability from N2 to W sleep was higher in non-COVID-related insomnia. One study revealed that individuals with insomnia are more likely to experience early termination of stage N2 sleep bouts and an increased likelihood of transitioning from stage N2 to stage N1 sleep or wakefulness [20]. In the latter study, changes in stage transition in insomnia versus healthy controls were investigated, marking N2 sleep vulnerability as a marker of insomnia. Additionally, we found markers indicating N3 (i.e. lower chance of transition to N3 and shorter duration of N3 bouts), and REM instability (i.e. higher chance of transitioning to N1 and shorter duration of bouts) as well as a longer onset to stable REM sleep. It is important to note that REM sleep, being the most highly aroused brain state during sleep, is particularly susceptible to fragmentation in individuals experiencing persistent hyperarousal [19]. Additionally, REM sleep fragmentation in insomnia patients is associated with anxiety; this could be a contributing factor in patients with post-COVID-19, where neuropsychiatric symptoms are common. Alteration in REM parameters could also suggest an additional circadian misalignment due to lockdown and lack of natural “Zeitgebers.” Notably, individuals with insomnia exhibited greater sleep fragmentation after the onset of the pandemic, regardless of whether they had been infected with COVID-19, than those with pre-pandemic insomnia, indicating an additional acute disruption tied to the pandemic.

Synthesizing these findings, insomnia can manifest with different “signatures” of sleep continuity disturbance. In stressful contexts such as a pandemic, heightened emotional, or physiological arousal may particularly disrupt REM, N3 sleep, or lead to increased sleep fragmentation, while longstanding or more typical forms of insomnia may place more emphasis on light sleep stability (N2). Larger studies are required to further understand these nuances and could guide tailored interventions, whether the focus is on stress/anxiety reduction or on other approaches to improve sleep continuity and reduce nighttime awakenings.

### REM sleep without atonia in post-COVID-19 patients

In our study, we found a lower RAI in post-COVID-19 insomnia patients. This extends our previous study, where 36 per cent of the patients exhibited RWA following acute COVID-19 infection [22]. The current findings provide further evidence of direct CNS involvement post-COVID-19. One recent study showed electrophysiological changes (EEG abnormalities) in long-COVID patients resembling those observed in the early stages of neurodegenerative diseases [47]. Additionally, in line with our findings, in a specialized post-COVID-19 center, Coelho et al. reported a high prevalence of 11.9 per cent of clinical RBD [9]. Quantitatively, our post-COVID-19 insomnia cohort showed a median RAI-Chin of 0.94 (IQR 0.89–0.97). Ferri et al. [30] reported mean atonia index values of 0.960 ± 0.022 in young controls, 0.920 ± 0.071 in untreated OSA, and 0.796 ± 0.164 in isolated RBD. The median score of our cohort, therefore, remains above the conventional 0.90 “normal” cut-off. However, the ~0.03-point decrement we observed mirrors the control-versus-OSA gap and may reflect a subtle COVID-related attenuation of REM atonia rather than full-blown neurodegeneration/clinical RBD. Longitudinal monitoring is warranted to determine whether this modest reduction is a benign epiphenomenon or an early marker that could accelerate neurodegenerative risk.

### Strengths and limitations

Our study has several strengths, including the use of level I laboratory polysomnography to assess sleep in insomnia patients, the careful matching of the cohorts, and the use of controls from two different time periods, i.e. pre-pandemic and during the pandemic. Limitations of our study include the retrospective design, which, however, in this case, was necessary to investigate possible pre- and post-pandemic changes. Since no pre-infection v-PSG was available, we cannot definitively confirm premorbid sleep normalcy. Nonetheless, all post-COVID-19 patients reported that their insomnia began only after the infection, and we compared them with age- and sex-matched insomnia patients without COVID-19. Additionally, the only subjective measure of sleep included was the ESS and while this did not differ significantly among groups (median score = 4 at the time of v-PSG), the v-PSG was performed at least 3 months after the acute phase; other sleep disturbances (e.g. hypersomnia) were not explicitly assessed during the acute period, even though such symptoms are commonly reported [48]. Implementing insomnia-specific scales (e.g. ISI) would have provided more insight into the subjective complaints. Finally, within the post-COVID-19 cohort, part of the sample was included in our previous study [22], and we included COVID-19 patients based on clinical history; antibody test was not routinely performed.

## Conclusions

In this polysomnographic study examining objective sleep changes in post-COVID-19 insomnia, we found no significant differences in classical polysomnographic parameters between post-COVID-19 insomnia patients and those with non-COVID-19-related chronic insomnia, both before and during the pandemic. This indicates a shared basic pathophysiologic mechanism underlying insomnia in both groups. However, a trend toward alterations in the sleep transition probabilities, increased sleep fragmentation, and higher heart rates of post-COVID-19 insomnia patients was observed, suggesting higher sleep disturbance and possible dysautonomia. Additionally, these patients exhibited increased REM sleep without atonia, a likely consequence after viral infection. These findings suggest that, other than the pandemic factors, post-COVID-19 insomnia presents additional sleep changes that are resulting from direct CNS involvement. Nevertheless, because the observed differences are minor, our findings support that the same management approach can be applied to post-COVID-19 insomnia. Future research is needed to determine whether infection-related factors in insomnia should guide more tailored treatment strategies.

## Supplementary Material

Sleep_Architecture_and_REM_Sleep_Without_Atonia_in_Pos_COVID_Supplementary_zsaf257

## Data Availability

The datasets generated and analyzed during the current study are available from the corresponding authors on reasonable request.
