# Supplementary material for: Sleep architecture and rapid eye movement sleep without atonia in post-COVID-19 insomnia
Source: Sleep. 2025 Sep 16;49(3):zsaf257. doi: 10.1093/sleep/zsaf257 (PMC13016631; doi:10.1093/sleep/zsaf257)
Supplement: Sleep_Architecture_and_REM_Sleep_Without_Atonia_in_Pos_COVID_Supplementary_zsaf257 [file sleep_architecture_and_rem_sleep_without_atonia_in_pos_covid_supplementary_zsaf257.docx]

**Sleep Architecture and REM Sleep Without Atonia in Post-COVID-19 Insomnia**

Abubaker Ibrahim^1^, Matteo Cesari^1^, Qi Tang^1^, Merve Aktan Süzgün^1^, Elisabeth Brandauer^1^, Evi Holzknecht^1^, Alexander Wachter^1^, Victoria Anselmi^1^, Anna Heidbreder^2^, Ambra Stefani^1^, Birgit Högl^1^

**Author affiliations:**

^1^Department of Neurology, Medical University of Innsbruck, Innsbruck, Austria

^2^Department of Neurology, Johannes Kepler University Linz, Linz, Austria.

Correspondence to: Birgit Högl

Full address: Medical University of Innsbruck, Department of Neurology, Anichstraße 35, 6020 Innsbruck, Austria

E-mail: [Birgit.ho@i-med.ac.at](mailto:Birgit.ho@i-med.ac.at)

Disclosures: None

**Supplementary materials**

# **Supplementary Table 1: Stage shifts and transition probabilities between sleep stages**

| Parameter | Post-COVID-19 insomnia = 50 | Non-COVID-related insomnia = 100 | Mann-Whitney | Adjusted *P* | Effect size |
| --- | --- | --- | --- | --- | --- |
| W🡪W | 85.5 (76.6-93.1) | 85.0 (80.7-90.1) | 0.677 | 0.767 | 0.03 |
| W🡪N1 | 13.5 (6.2-23.1) | 14.4 (9.4-19.1) | 0.586 | 0.712 | 0.04 |
| W🡪N2 | 0 (0-0.0) | 0 (0-0.4) | **-** | - | - |
| W🡪N3 | 0 (0-0) | 0 (0-0) | - | - | - |
| W🡪REM | 0 (0-0) | 0 (0-0) | - | - | - |
| N1🡪W | 8.0 (4.3-11.9) | 9.2 (5.2-12.5) | 0.283 | 0.523 | 0.09 |
| N1🡪N1 | 56.2 (47.3-67.9) | 56.4 (46.8-62.4) | 0.357 | 0.523 | 0.08 |
| N1🡪N2 | 27.6 (21.6-38.0) | 32.1 (24.4-38.9) | 0.222 | 0.523 | 0.10 |
| N1🡪N3 | 0 (0-0) | 0 (0-0) | - | - | - |
| N1🡪REM | 2.6 (0-6.0) | 1.6 (0-3.8) | 0.203 | 0.523 | 0.10 |
| N2🡪W | 2.0 (1.1-2.7) | 2.4 (1.7-3.6) | **0.007** | 0.118 | 0.22 |
| N2🡪N1 | 2.8 (1.1-4.6) | 2.6 (1.5-4.3) | 0.883 | 0.883 | 0.01 |
| N2🡪N2 | 93.1 (90.9-95.3) | 92.3 (90.3-94.3) | 0.400 | 0.523 | 0.07 |
| N2🡪N3 | 1.2 (0.6-1.9) | 1.1 (0.6-1.6) | 0.270 | 0.523 | 0.09 |
| N2🡪REM | 0 (0-0) | 0 (0-0) | - | - | - |
| N3🡪W | 1.0 (0.0-1.9) | 1.2 (0.5-2.2) | 0.851 | 0.523 | 0.08 |
| N3🡪N1 | 0 (0-1.0) | 0 (0-0.6) | 0.188 | - | 0.11 |
| N3🡪N2 | 2.3 (1.2-3.3) | 1.7 (0.7-2.9) | 0.221 | 0.523 | 0.11 |
| N3🡪N3 | 95.8 (94.4-97.1) | 96.5 (94.8-97.3) | 0.178 | 0.523 | 0.12 |
| N3🡪REM | 0 (0-0) | 0 (0-0) | - | - | - |
| REM🡪W | 3.1 (1.5-4.6) | 2.3 (1.1-4.0) | 0.167 | 0.523 | 0.11 |
| REM🡪N1 | 1.3 (0-3.4) | 0.7 (0-2.0) | 0.071 | 0.523 | 0.15 |
| REM🡪N2 | 0.0 (0-0.9) | 0.5 (0-1.1) | **0.027** | - | 0.18 |
| REM🡪N3 | 0 (0-0) | 0 (0-0) | - | - | - |
| REM🡪REM | 95.3 (92.3-96.6) | 95.6 (93.4-96.9) | 0.308 | 0.523 | 0.08 |

**Legend:**

The values are expressed as median and interquartile range and represent the probability of transitioning between sleep stages.The same stage sleep transition indirectly reflects the stability of a certain sleep stage. In bold, the transitions, which are significant before multiple comparison corrections, are highlighted. After multiple comparison correction, none of the comparisons remained significant.

Abbreviations: COVID: coronavirus disease; ES: effect size; REM: rapid eye movement; W: wake

# **Supplementary Table 2: Onset to stable sleep and sleep bouts**

| Parameter | | COVID-19 insomnia,  N= 50 | Non-COVID-related insomnia, N= 100 | *P* Value | Effect size |
| --- | --- | --- | --- | --- | --- |
| Onset to stable sleep (10min) after light off, min | 39.8 (22.0- 57.8) | | 37.2 (20.8- 59.9) | 0.695 | 0.03 |
| Onset to stable sleep after SOL (10min), min | 19.2 ( 8.8- 39.1) | | 17.2 ( 5.9- 33.5) | 0.533 | 0.05 |
| Onset to stable N2 (10min) after SOL, min | 39.5 (13.2- 94.8) | | 8.2 ( 4.8- 17.5) | 0.237 | 0.10 |
| Onset to stable N3 (10min) after SOL, min | 36.5 (16.8- 70.5) | | 28.0 ( 17.8- 42.0) | 0.917 | 0.01 |
| Onset to stable REM (10min) after SOL, min | 142.8 (88.4-210.0) | | 190.0 (115.5-283.5) | **0.016** | 0.20 |
| Number of N1 bouts | 36.0 (26.0- 48.2) | | 34.5 (27.0- 47.5) | 0.741 | 0.03 |
| Number of N2 bouts | 28.5 (22.0- 39.2) | | 28.0 (20.0- 35.0) | 0.496 | 0.06 |
| Number of N3 bouts | 4.0 ( 3.0- 6.0) | | 5.0 ( 3.0- 7.0) | 0.612 | 0.04 |
| Number of REM bouts | 6.0 ( 4.0- 9.0) | | 7.0 ( 5.0- 10.0) | 0.225 | 0.10 |
| Number of Wake bouts | 24.0 (18.8- 33.0) | | 21.5 (15.0- 31.0) | 0.135 | 0.12 |
| Longest N1 duration, min | 4.0 ( 3.0- 5.1) | | 5.0 ( 3.0- 6.5) | 0.066 | 0.15 |
| Longest N2 duration, min | 27.0 (20.9- 35.6) | | 30.5 (23.6- 36.9) | 0.347 | 0.08 |
| Longest N3 duration, min | 27.2 (22.0- 35.1) | | 23.0 (16.8- 32.5) | 0.067 | 0.16 |
| Longest REM duration, min | 24.5 (19.0- 33.9) | | 20.5 (16.0- 32.5) | 0.086 | 0.14 |
| Longest wake duration, min | 30.0 (19.2- 51.6) | | 29.8 (15.0- 53.5) | 0.658 | 0.04 |
| Average N1 bout duration, min | 1.1 ( 0.9- 1.3) | | 1.1 ( 0.9- 1.6) | 0.347 | 0.08 |
| Average N2 bout duration, min | 6.5 ( 5.1- 8.7) | | 7.2 ( 5.5- 10.5) | 0.411 | 0.07 |
| Average N3 bout duration, min | 14.2 ( 9.7- 18.0) | | 12.0 ( 9.0- 17.2) | 0.184 | 0.11 |
| Average REM bout duration, min | 11.2 ( 7.6- 16.3) | | 10.6 ( 6.5- 14.8) | 0.322 | 0.08 |
| Average Wake bout duration, min | 3.2 ( 2.5- 4.9) | | 3.4 ( 2.1- 6.8) | 0.671 | 0.03 |
| Sleep fragmentation Index (Haba Rubio)/h | 16.2 (12.4- 21.6) | | 15.3 ( 12.4- 19.3) | 0.435 | 0.06 |
| Sleep fragmentation Index (Morrel)/h | 9.1 ( 6.8- 13.7) | | 8.8 ( 6.7- 11.5) | 0.354 | 0.08 |
| Count of transitions to different sleep stages | 100.0 (77.8-131.2) | | 94.0 ( 74.2-131.0) | 0.574 | 0.05 |

**Legend:**

The values are expressed as median and interquartile range. In bold, the comparisons, which are significant before multiple comparison corrections, are highlighted. Onset to stable N1 sleep is not displayed since the median of participants with stable N1 sleep ≥10 minutes was 0.

Abbreviations: REM: rapid eye movement; COVID: coronavirus disease; SOL: sleep onset

**Supplementary Figure 1. Sleep stage transition probability**

1. Pre-pandemic insomnia,


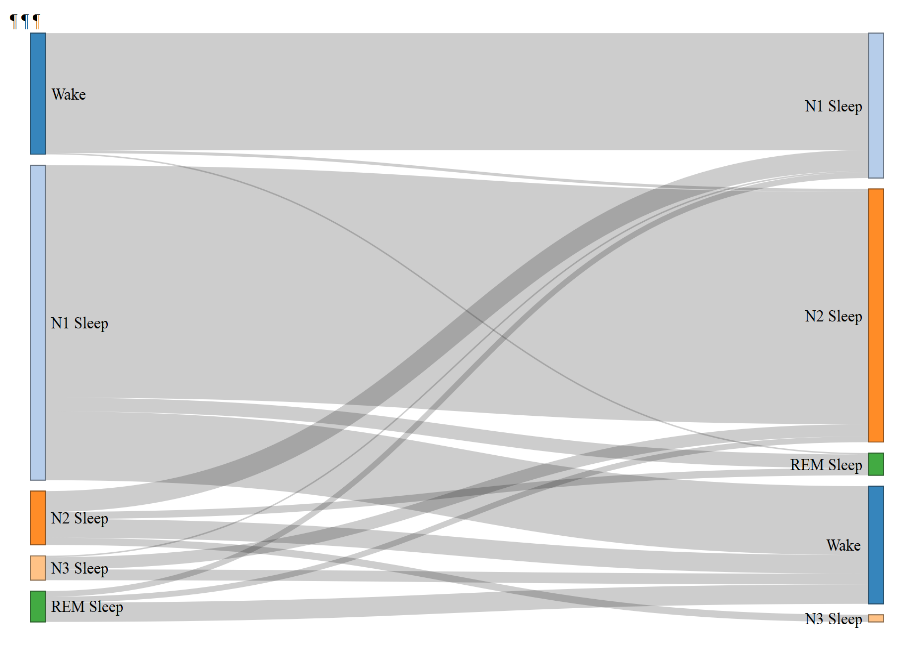

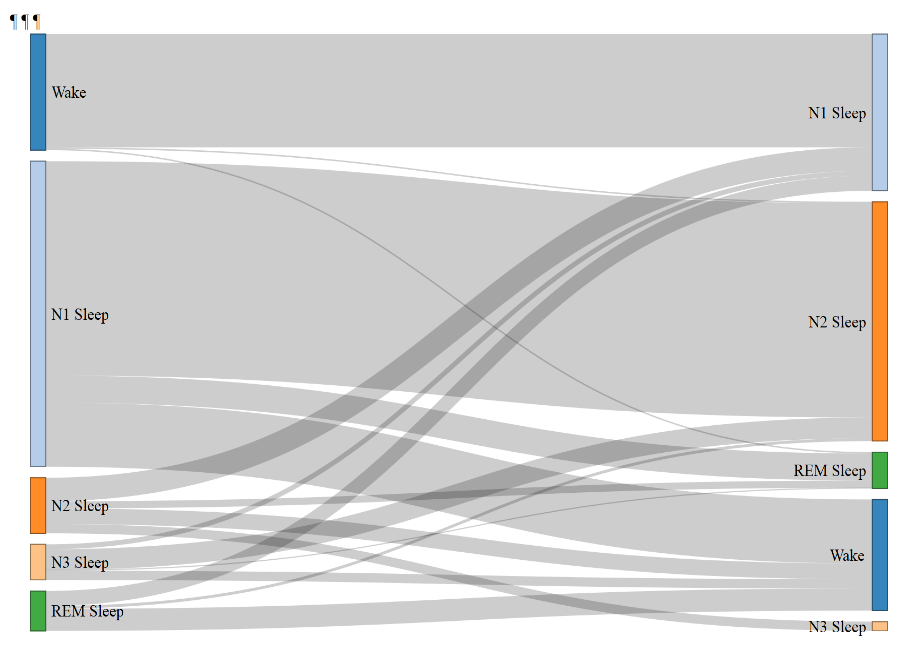


1. Post-COVID-19 Insomnia


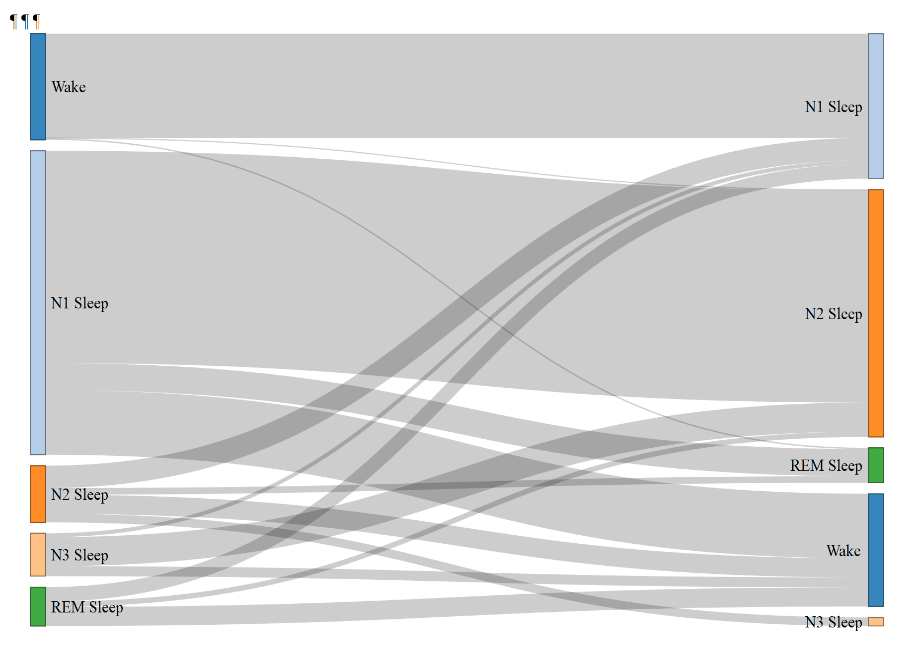


1. Pandemic-era, non-COVID insomnia

**Legends:**

Sankey's plot demonstrates the mean transition probability from one sleep stage to the next (epoch>epoch) within the three groups. For better visuality, the same stage transition (e.g., N1->N1) was removed

Abbreviations COVID: coronavirus disease; REM, rapid eye movement
